# A naturally occurring bovine APOBEC3 confers resistance to bovine lentiviruses: implication for the co-evolution of bovids and their lentiviruses

**DOI:** 10.1038/srep33988

**Published:** 2016-09-26

**Authors:** Eri Yamada, Rokusuke Yoshikawa, Yusuke Nakano, Naoko Misawa, Tomoko Kobayashi, Fengrong Ren, Taisuke Izumi, Takayuki Miyazawa, Yoshio Koyanagi, Kei Sato

**Affiliations:** 1 Laboratory of Viral Pathogenesis, Institute for Virus Research, Kyoto University, Kyoto 6068507, Japan; 2 Laboratory of Animal Health, Department of Animal Science, Faculty of agriculture, Tokyo University of Agriculture, Kanagawa 2430034, Japan; 3 Department of Bioinformatics, Medical Research Institute, Tokyo Medical and Dental University, Tokyo 1138510, Japan; 4 CREST, Japan Science and Technology Agency, Saitama 3220012, Japan; 5 Laboratory of Virolution, Institute for Virus Research, Kyoto University, Kyoto 6068507, Japan

## Abstract

Mammals have co-evolved with lentiviruses for a long time. As evidence, viral infectivity factor (Vif), encoded by lentiviruses, antagonizes the anti-viral action of cellular APOBEC3 of their hosts. Here, we address the co-evolutionary dynamics of bovine APOBEC3 and the following two bovine lentiviruses: bovine immunodeficiency virus (BIV) and Jembrana disease virus (JDV). We determined the sequences of three *APOBEC3* genes of bovids belonging to the genera *Bos* and *Bison* and showed that bovine *APOBEC3Z3* is under a strong positive selection. We found that APOBEC3Z3 of gaur, a bovid in the genus *Bos*, acquired resistance to JDV Vif-mediated degradation after diverging from the other bovids through conversion of the structural composition of the loop 1 domain. Interestingly, the resistance of gaur APOBEC3Z3 can be attributed to the positive selection of residue 62. This study provides the first evidence, suggesting that a co-evolutionary arms race between bovids and lentiviruses occurred in Asia.

During the co-evolution of viruses and their hosts, new lineages of viruses can be emerged through cross-species viral transmission^1^,^2^. However, intrinsic cellular proteins can be a hurdle to impairing cross-species viral transmission^3^. Human apolipoprotein B mRNA editing enzyme catalytic polypeptide-like 3 (APOBEC3; A3) proteins, particularly APOBEC3G (A3G), are cellular DNA cytosine deaminases and one of the well-studied restriction factors that inhibit the replication of human immunodeficiency virus type 1 (HIV-1), the causative agent of acquired immunodeficiency syndrome (AIDS) in humans^4^. A3 proteins are packaged into released HIV-1 virions and insert G-to-A hypermutation in newly synthesized viral cDNA. Such A3-mediated hypermutation results in the termination of viral replication. To exclude the A3-mediated anti-viral effect, an HIV-1-encoded protein, viral infectivity factor (Vif), recruits a cellular E3 Ubiquitin ligase, Cullin 5, and certain co-factors such as Elongin B/C (ELOGB/C)^4^,^5^. The Vif-mediated E3 ubiquitin ligase complex impedes the incorporation of A3 proteins into viral particles by degrading A3 proteins via the ubiquitin/proteasome pathway^4^,^5^.

Rodents encode a sole *A3* gene, while primates including humans encode 7 paralogs designated *A3A*-*H* (*A3A, A3B, A3C, A3D, A3E, A3F,* and *A3H*)^4^. Given that positive Darwinian selection contributes to gene duplication, these *A3* genes are positively selected during primate evolution^6^. Importantly, primate lentiviral Vif antagonizes primate anti-viral A3 proteins in a species-specific manner^7^,^8^, suggesting that the specificity of lentiviral Vif reflects the adaptation episode of primate lentiviruses to their hosts. Moreover, the relationship between host proteins (e.g., A3) and viral proteins (e.g., Vif) can shed light on the co-evolutionary history of hosts and viruses and/or their evolutionary arms race^6^,^9^,^10^. As *A3G* is highly diversified in Old World monkeys in Africa, the specificity of Vif reflects the lineage of primate lentiviruses^9^,^11^,^12^. These previous observations provide a possibility that revealing the specificity of lentiviral Vif can be a clue to interpret the evolutionary history of lentivirus infection in the past^9^.

Bovids including cattle (*Bos taurus*), belong to the *Bovini* tribe, which is composed of the genera *Bos, Bison,* and *Bubalus*^13^,^14^,^15^. Cattle, zebu (*Bos indicus*), banteng (*Bos javanicus*), gaur (*Bos gaurus*), and yak (*Bos grunniens*) belong to the genus *Bos*, while American bison (*Bison bison*) and European bison (*Bison bonasus*) belong to the genus *Bison.* In contrast to rodents and primates, ruminants, including cattle, encode the following 3 *A3* genes: *A3Z1, A3Z2,* and *A3Z3*^16^,^17^. Cattle also express another splicing variant, *A3Z2Z3,* and a previous study has reported that cattle A3Z3 and A3Z2Z3 exhibit a strong anti-lentiviral ability in *in vitro* cell culture systems^17^. However, the *A3* sequences of other bovids have not yet been determined.

In bovids, two lentiviruses have been reported to date. One is bovine immunodeficiency virus (BIV), while the other is Jembrana disease virus (JDV). Since 1964, sporadic outbreaks, resulting in severe disorders and high mortality, have occurred in Bali cattle, known as domesticated banteng, in certain islands in Indonesia^18^. In 1993, JDV was isolated and identified as the causative agent of severe disorders in Bali cattle^19^. The JDV infection is endemic in Bali cattle, banteng, and cattle in the islands of Southeast Asia, including Indonesia and Malaysia^18^,^19^,^20^. BIV was isolated in 1972 in the United States from cattle displaying persistent lymphocytosis^21^. Subsequent studies have characterized this virus as a lentivirus, named BIV because of its genetic relationship and biological similarity to HIV-1^22^,^23^. However, the disorders caused by a BIV infection in cattle seem relatively mild compared to those that occur in humans as a result of HIV-1 infection^24^,^25^,^26^. In contrast to JDV, BIV infections have been reported worldwide, and serosurveillance studies have reported that BIV infections have been found in Europe^27^,^28^,^29^, North America^21^,^30^, South America^31^, Africa^32^, and Asia^33^,^34^.

As described above, the co-evolutionary dynamics and/or evolutionary arms race between primates and their lentiviruses, particularly primate A3 and their lentiviral Vif, have been well documented^6^,^9^,^10^,^11^,^12^. However, although the evolutionary interplay between mammalian A3 and lentiviral Vif can elucidate their co-evolutionary history, the co-evolutionary relationship between non-primate mammals and their lentiviruses remains unclear.

In this study, we focus on the co-evolutionary dynamics of bovids and bovine lentiviral Vif proteins. We determine the sequences of the three *APOBEC3* genes in the genera *Bos* and *Bison* and show that bovine *A3Z3* is under a strong positive selection. We conduct a combination of studies consisting of molecular phylogenetics, structural biology, and experimental virology. Our findings provide evidence, suggesting that a co-evolutionary arms race between bovids and lentiviruses has occurred in Asia.

## Results

### Strong positive selection detected in bovine *A3Z3*

**Figure 1:**
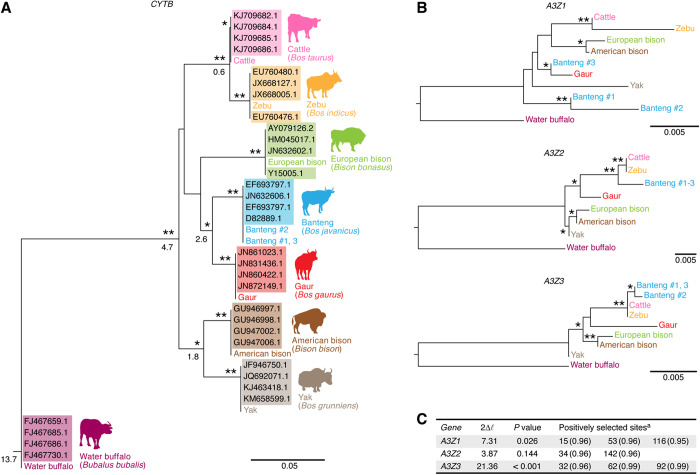
Molecular phylogenetic of bovine *CYTB* and *A3* genes. (**A**) Phylogenic tree of bovine *CYTB*. The sequences of bovine *CYTB,* which were identified from the samples used in this study, were aligned with four *CYTB* sequences of respective species deposited in GenBank (the accession numbers are shown with shading), and the tree was reconstructed using the ML method. The numbers under certain nodes indicate the age of divergence (million years ago) that was estimated in a previous study^14^. (**B**) Phylogenic trees of bovine *A3*. The sequences of bovine *A3Z1* (top), which were newly identified in this study, were aligned, and the trees were reconstructed using the ML method. Note that the *A3* sequences of cattle are identical to those reported in a previous study^16^. In (**A**,**B**) the sequences of water buffalo were used as the outgroup. Nodes with higher bootstrap support are denoted by asterisks as follows: *, 0.50%; **, 0.80%. The scale bar indicates an evolutionary distance of 0.05 (**A**) and 0.005 (**B**) nucleotide substitutions per site, respectively. (**C**) Positive selection of bovine *A3*. Parameters were estimated under two pairs of site models in the PAML package. Positive selection was detected for bovine *A3Z3* at a high significance level (*P* < 0.001). The positively selected sites are listed. The values in parentheses indicate posterior probability. The dN/dS ratio and posterior probability inferred for each codon position by PAML analysis are summarized in Supplementary Tables 1–3. 2Δ*l*, twice the log likelihood difference between the two compared models.

In order to investigate the co-evolutionary relationship between bovids and their lentiviruses, we collected tissue samples from the following seven species belonging to the *Bovini* tribe (the genera *Bos*, *Bison,* and *Bubalus*): cattle (*Bos taurus*), zebu (*Bos indicus*), banteng (*Bos javanicus*), gaur (*Bos gaurus*), yak (*Bos grunniens*), American bison (*Bison bison*), European bison (*Bison bonasus*) and water buffalo (*Bubalus bubalis*; belonging to the genus *Bubalus*) from Japanese zoos. By analyzing the sequences of bovine cytochrome b gene (*CYPB*) and comparing them to those deposited in the database, we confirmed that our samples are derived from these species (Fig. 1A). We then sought to determine the sequences of the following three bovine *A3* genes: *A3Z1, A3Z2,* and *A3Z3* (Table 1; see also Supplementary Figure 1). In this sequencing analysis, we detected putative interferon-sensitive responsive elements (ISREs) upstream of exon 1 of bovine *A3Z2* and *A3Z3* (Supplementary Figure 2), suggesting that the expression of bovine *A3* genes is regulated in a similar manner.

Next, we constructed open reading frames (ORFs) of bovine *A3* genes by conjugating exon sequences and aligning them with reconstructed phylogenetic trees consisting of these genes. As shown in Fig. 1B, the topologies of the phylogenic trees of respective bovine *A3* genes were different from one another. Additionally, these topologies were incongruent with the species phylogeny determined by *CYPB* (Fig. 1B). These findings raise at least two possibilities as follows: 1) the classification of bovine *A3* genes into *A3Z1, A3Z2,* and *A3Z3* is not reliable; or 2) bovine *A3* genes are under positive selection and have had a complex evolutionary history as in the case of primate *A3* genes^12^. To address the former possibility, we reconstructed an unrooted phylogenetic tree using all bovine *A3* genes. As shown in Supplementary Figure 2, bovine *A3Z1*, *A3Z2* and *A3Z3* formed a single cluster with 100% bootstrap support. This result argues against the possibility that bovine *A3* genes are not sorted correctly. To test the latter possibility of a positive selection of respective bovine *A3* genes, we performed molecular phylogenetic analyses and revealed that bovine *A3Z1* and *A3Z3* are under positive selection (Fig. 1C; the raw results are summarized in Supplementary Tables 1–3). Particularly, we found that bovine *A3Z3* is likely under strong positive selection (*P* < 0.001) and that the three amino acids positioned at 32, 62, and 92 are positively selected (Fig. 1C).

**Figure 2:**
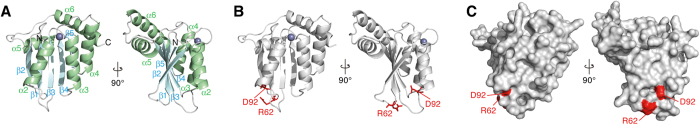
Structure modeling of bovine A3Z3. Cartoon (**A**,**B**) and surface (**C**) models of the structure of cattle A3Z3 are shown. In (**A**) α-helices and β-sheets are shown in green and pale blue, respectively. In (**A**,**B**) Zn^2+^ is represented as a gray sphere. In (**B**,**C**) the positively selected amino acids (residues 62 and 92) are represented in red. Note that the α1 domain, including residue 32, is not shown, because residues 1–54 were not reconstructed by structure homology modeling.

To assess the position of the three positively selected sites, we constructed a homology model of cattle A3Z3 based on the crystal structure of the Vif binding domain of human A3F^35^. Although the Z domain of human A3 proteins^35^,^36^ and feline A3Z3, which we previously modeled^37^, possess 6 α-helices, cattle A3Z3 is predicted to be composed of 5 α-helices (α2 to α6) and 5 β-sheets (β1 to β5) (Fig. 2A). Because the structure of residues 1–54 was unable to be simulated, this model lacks the putative α1 domain. Nevertheless, the structure of cattle A3Z3 was generally similar to those of human A3^35^,^36^ and feline A3Z3 (Fig. 2A)^37^. We then plotted the positions of the positively selected residues (Fig. 1C) on our homology model. Residue 62 was appeared to be located between β1 and β2, while the residue 92 was positioned at the end of the α2 domain (Fig. 2B). Moreover, as shown in Fig. 2C, residues 62 and 92 were predicted to be localized on the surface of the protein.

### Resistance of gaur A3Z3 to JDV Vif-mediated degradation

**Figure 3:**
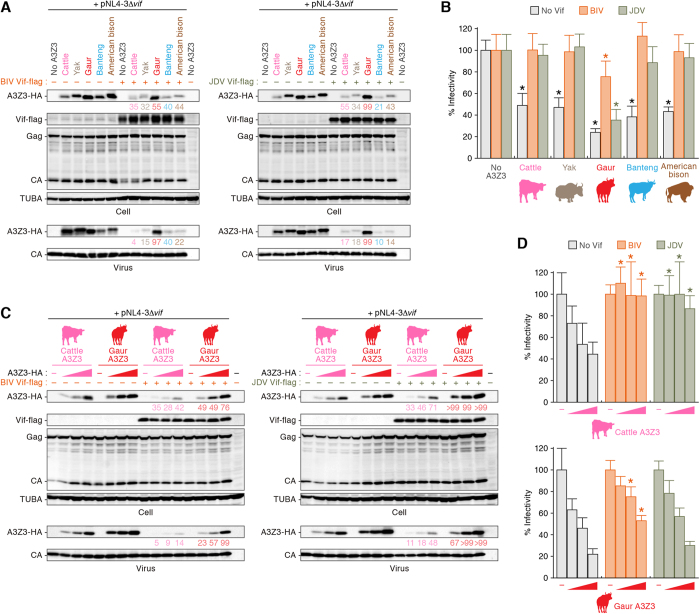
Resistance of gaur A3Z3 to BIV/JDV Vif-dependent degradation. (**A**,**B**) Anti-lentiviral ability of bovine A3Z3 proteins. The HA-tagged expression plasmids for A3Z3 of cattle, yak, gaur, banteng, and American bison (100 ng) and pNL4-3Δ*vif* (*vif-*deleted HIV-1-producing plasmid; 700 ng) were co-transfected with the expression plasmid for either flag-tagged BIV Vif or JDV Vif (1,000 ng). (**A**) Western blotting. Representative results are shown. Note that zebu and European bison A3Z3 were not used in this experiment because their amino acid sequences are identical to cattle and American bison A3Z3, respectively (also see Fig. 5A). (**B**) TZM-bl assay. Viral infectivity is shown as the percentage of the value of “no A3Z3.” Asterisks represent *P* < 0.05 (versus “no A3Z3” by Student’s *t* test). The assays were independently performed in triplicate. The data represent averages with SD. (**C**,**D**) Sensitivity of cattle and gaur A3Z3 to BIV/JDV Vif-mediated degradation. The HA-tagged expression plasmids for cattle or gaur A3Z3 (0, 25, 50, and 100 ng) and pNL4-3Δ*vif* (*vif-*deleted HIV-1-producing plasmid; 700 ng) were co-transfected with the flag-tagged expression plasmids for either BIV Vif or JDV Vif (1,000 ng). (**C**) Western blotting. Representative results are shown. (**D**) TZM-bl assay. Viral infectivity is shown as the percentage of the value of no A3Z3 (“–” in the figure). Asterisks represent *P* < 0.05 (versus the value of “no Vif” for the same amount of A3Z3 by Student’s *t* test). The assays were independently performed in triplicate. The data represent averages with SD. In (**A**,**C**) the value under the band of respective bovine A3Z3-HA represents the percentage of band intensity compared to the absence of Vif. Blots have been cropped; uncropped blots are shown in Supplementary Figure 6.

To elucidate the relationship between bovine A3Z3 and the counteracting ability of bovine lentiviral Vif proteins, we co-transfected expression plasmids for bovine A3Z3 and the Vif proteins of BIV or JDV. In the absence of bovine lentiviral Vif, the expression levels of bovine A3Z3 proteins were similar, and the incorporation efficacies of these A3Z3 proteins into viral particles were comparable (Fig. 3A). In addition, the infectivity of *vif*-deleted virions was significantly suppressed by all bovine A3Z3 proteins at comparable levels (Fig. 3B). We then assessed the anti-viral activity of bovine A3Z3 in the presence of bovine lentiviral Vif. As shown in Fig. 3A, although all bovine A3Z3 proteins, with the exception of gaur A3Z3, were degraded in a bovine lentiviral Vif-dependent manner, gaur A3Z3 was surprisingly partially resistant to BIV Vif-mediated degradation and almost completely resistant to JDV Vif-mediated degradation. Moreover, even in the presence of bovine lentiviral Vif, gaur A3Z3 was efficiently packaged into the released virions (Fig. 3A) and was able to suppress viral infectivity (Fig. 3B). In particular, gaur A3Z3 exhibited strong suppression of viral infectivity in the presence of JDV Vif but only partially decreased viral infectivity in the presence of BIV Vif.

To determine whether the expression level of bovine A3Z3 affected this observation, we transfected the expression plasmids for cattle and gaur A3Z3 at 3 different doses. As shown in Fig. 3C,D, both BIV and JDV Vif degraded cattle A3Z3, and the viral infectivity was completely recovered independently of the expression level of cattle A3Z3. In contrast, although BIV Vif partially degraded gaur A3Z3, the resistance of gaur A3Z3 to JDV Vif-dependent degradation was maintained regardless of the expression level of gaur A3Z3 (Fig. 3C,D).

As described in the Introduction, cattle express another splice variant of A3, called A3Z2Z3^17^. To further address the possibility that bovine A3Z2Z3 including gaur A3Z2Z3, is resistant to degradation mediated by BIV or JDV Vif, we prepared a series of bovine A3Z2Z3 expression plasmids, which were co-transfected with the Vif expression plasmids. However, we found that all bovine A3Z2Z3 proteins, including gaur A3Z2Z3, were efficiently degraded by both BIV and JDV Vif (Supplementary Figure 5). Altogether, these findings suggest that only gaur A3Z3 is resistant to JDV Vif-mediated degradation and elicits an anti-viral ability even in the presence of JDV Vif.

### Residues 57–102 in bovine lentiviral Vif determine its ability to degrade gaur A3Z3

**Figure 4:**
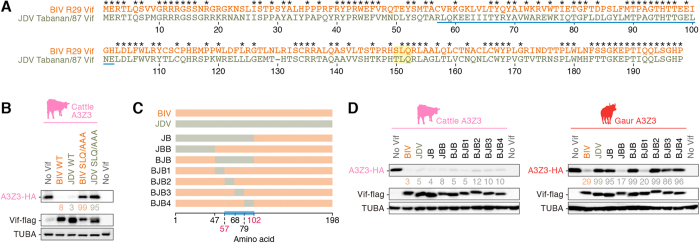
Region of bovine lentiviral Vif responsible for degradation of gaur A3Z3. (**A**) Alignment of the amino acid sequences of BIV and JDV Vif. Asterisks indicate the identical amino acid. The determined responsible region is indicated by a blue line (residues 57–102; identical to that in panel C). The S/TLQ motif is indicated by yellow shading (residues 150–152). (**B**) Western blotting. The HA-tagged expression plasmid for cattle A3Z3 (150 ng) was co-transfected with the flag-tagged expression plasmids for wild-type (WT) or SLQ/AAA-mutated BIV/JDV Vif (1,000 ng). (**C**,**D**) Exchanging derivatives of BIV and JDV Vif. (**C**) Scheme of a series of exchanging mutants. The names of respective derivatives (indicated on the left of the panel) are identical to those in panel D. The determined responsible region is indicated by a blue line (residues 57–102; identical to that in panel A). (**D**) Western blotting. The HA-tagged expression plasmids for cattle or gaur A3Z3 (150 ng) were co-transfected with the flag-tagged expression plasmids for a series of BIV/JDV Vif derivatives (1,000 ng). In panels B and D, the value under the band of each bovine A3Z3-HA represents the percentage of band intensity compared to the absence of Vif. Blots have been cropped; uncropped blots are shown in Supplementary Figure 8.

Although the crystal structure of HIV-1 Vif has been identified^38^, the amino acid sequence of bovine lentiviral Vif is divergent from that of the other lentiviral Vifs, including HIV-1 Vif^39^,^40^. Therefore, it is technically impossible to simulate the tertiary structure of bovine lentiviral Vif based on HIV-1 Vif. Moreover, it has been reported that BIV Vif utilizes Cullin 2, a cellular E3 Ubiquitin ligase, to degrade cattle A3Z3^41^,^42^, while HIV-1 Vif utilizes Cullin 5, another E3 Ubiquitin ligase^4^,^5^. Interestingly, the S/TLQ motif, which is responsible for the binding to Elongin B/C leading to the orchestration of cellular E3 Ubiquitin ligase complex^4^,^5^, is conserved in both BIV and JDV Vif (Fig. 4A, indicated by yellow shading). To test whether bovine lentiviral Vif degrades bovine A3Z3 in an S/TLQ motif-dependent manner (i.e., similarly to HIV-1 Vif), we prepared expression plasmids for BIV and JDV Vif in which S/TLQ residues were mutated to alanines. As shown in Fig. 4B, S/TLQ → AAA mutants completely abolished the degradation, suggesting that both BIV and JDV Vif degrade bovine A3Z3 in an S/TLQ motif-dependent manner.

Next, we determined which residues were responsible for the degradation of gaur A3Z3 by bovine lentiviral Vif. Because the homology of BIV Vif to JDV Vif is relatively low (Fig. 4A; amino acid identity, 55.1%; amino acid similarity, 83.8%. See also Supplementary Figure 4), we prepared a series of swapping derivatives (Fig. 4C). As shown in Fig. 4D (left panel), all BIV/JDV Vif derivatives, BIV Vif and JDV Vif degraded cattle A3Z3. Under these conditions, we revealed that the JBB and BJB1 derivatives degraded gaur A3Z3, while the others (JB, BJB, BJB2-4) were incapable of degrading gaur A3Z3 (Fig. 4D, right panel). These findings suggest that multiple amino acid residues, which are broadly localized in within residues 57–102 (indicated by horizontal blue bars in Fig. 4B,D), determine the ability to degrade gaur A3Z3.

### Evolutionary and structural insights into how gaur A3Z3 acquired resistance to JDV Vif-mediated degradation

**Figure 5:**
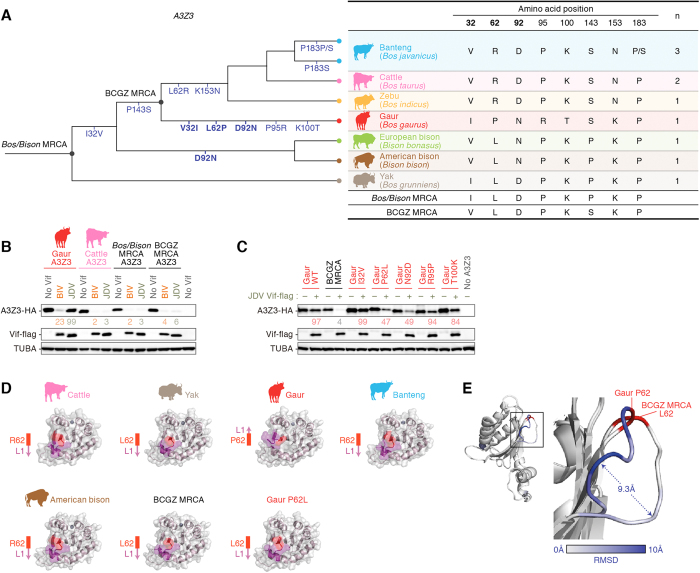
Evolutionary and structural insights into the acquisition of the resistance to JDV Vif-mediated degradation by gaur A3Z3. (**A**) Scheme of the evolution of bovine A3Z3. (Left) Phylogenic tree of bovine *A3Z3.* The acquired nonsynonymous mutations are indicated under each branch in blue. The three amino acids under positive selection (positioned at 32, 62, and 92; see also Fig. 1C) are indicated in bold. The two nodes of interest, *Bos/Bison* MRCA and BCGZ MRCA, are indicated by black dots. (Right) Summary of the amino acids of A3Z3 showing the differences among the genera *Bos* and *Bison.* The amino acid residues at the indicated positions of respective bovid as well as *Bos/Bison* MRCA and BCGZ MRCA are summarized. The three amino acid positions under positive selection (positioned at 32, 62, and 92) are indicated in bold. “n” is the number of sequences analyzed. Note that the amino acid sequence of cattle A3Z3 determined in this study (see also Fig. 1B) is identical to that of cattle A3Z3 deposited in GenBank (accession no. EU864536). (**B**,**C**) Western blotting. The HA-tagged expression plasmids for the different A3Z3 proteins (150 ng) were co-transfected with the flag-tagged expression plasmids for either BIV Vif or JDV Vif (1,000 ng). (**D**,**E**) Structural insights into bovine A3Z3. (**D**) Structure modeling of bovine A3Z3. Cartoon and surface models are overlaid. The amino acid positioned at 62 (L, R, or P) is indicated in red, and the L1 (NDLT; residues 63–66) is show in purple. The direction of the L1 loop is indicated by a purple arrow to the left of each model. (**E**) Overlay of BCGZ MRCA and gaur A3Z3. The boxed area in the L1 region (left) is enlarged to the right. The amino acid positioned at 62 (P in gaur, L in BCGZ MRCA) is indicated in red, and the RMSD value is shown in gray-to-blue (scale is shown under the panel). The distance of the Cα in L1 of gaur A3Z3 and BCGZ MRCA A3Z3 is indicated by double-headed arrow. In (**B**,**C**) the value under the band of each bovine A3Z3-HA represents the percentage of band intensity compared to the absence of Vif. Blots have been cropped; uncropped blots are shown in Supplementary Figure 9.

To determine when and how gaur A3Z3 acquired resistance to JDV Vif-mediated degradation, we evaluated the bovine *A3Z3* phylogenic tree (Fig. 5A). Based on this phylogenic tree, we estimated the sequences of the two *A3Z3* most recent common ancestors (MRCAs), including MRCAs of banteng, cattle, gaur, and zebu (BCGZ MRCA) and MRCAs of all of the genera *Bos* and *Bison* (*Bos/Bison* MRCA) (indicated by black dots in Fig. 5A). We prepared expression plasmids for these two MRCAs and co-transfected these plasmids with the bovine lentiviral Vif expression plasmids. As shown in Fig. 5B, both BCGZ MRCA and *Bos/Bison* MRCA were expressed at levels comparable to those of cattle and gaur A3Z3 in the absence of bovine lentiviral Vif. Although gaur A3Z3 was resistant to JDV Vif-mediated degradation (Fig. 5B), we found that these two MRCAs were degraded by Vifs of both BIV and JDV in a manner similar to that of cattle A3Z3 (Fig. 5B). These findings suggest that the resistance of gaur A3Z3 to JDV Vif-mediated degradation was evolutionary acquired after divergence from BCGZ MRCA.

We also inferred from the *A3Z3* phylogenic tree that gaur A3Z3 acquired 5 nonsynonymous mutations (V321I, L62P, D92N, P95R, and K100T) after divergence from BCGZ MRCA (Fig. 5A). To determine the residues of gaur A3Z3 responsible for the resistance to JDV Vif-mediated degradation, we prepared a series of point mutations based on the gaur A3Z3 sequence. As shown in Fig. 5C, in addition to parental gaur A3Z3, gaur A3Z3 mutants I32V, R95P, and T100K were resistant to JDV Vif-mediated degradation, while P62L and N92D gaur A3Z3 derivatives were degraded by JDV Vif. These results suggest that at least two amino acid residues, P62 and N92, of gaur A3Z3 are closely associated with the resistance to JDV Vif-mediated degradation compared to the other three residues.

As shown in Fig. 5A, the asparagine residue at 92 (N92) is encoded not only by gaur A3Z3 but also by the A3Z3 of European and American bison; only gaur A3Z3 has a proline at position 62. Therefore, to better understand how gaur A3Z3 acquired resistance to JDV Vif-mediated degradation, we focused on residue 62 and prepared homology models of A3Z3 of cattle, yak, gaur, banteng, American bison, and BCGZ MRCA, as well as a gaur P62L mutant (Fig. 5D). Although the amino acid residue positioned at 62 (R, L, or P) did not affect the position and direction of the residue itself, the direction of loop 1 (L1), which is composed of the residues 63–66 (NDLT), of gaur A3Z3 was opposite to those of cattle, yak, banteng, American bison, and BCGZ MRCA A3Z3 proteins (Fig. 5D). Interestingly, we also found that the direction of L1 of gaur P62L A3Z3 was similar to those of cattle, yak, banteng, American bison, and BCGZ MRCA A3Z3 proteins (i.e., opposite to that of parental gaur A3Z3) (Fig. 5D). To better elucidate this difference, the cartoon model of gaur A3Z3 was overlaid with that of BCGZ MRCA. As shown in Fig. 5E, we revealed that the L1 loop of gaur A3Z3 is differed from that of BCGZ MRCA by 9.3 Å, although the positions of each residue 62 (P for gaur, L for BCGZ MRCA, respectively) are similar to one another. Taken together, these findings suggest that gaur A3Z3 has acquired resistance to JDV Vif-mediated degradation by converting the direction of L1.

## Discussion

In this study, we determined the sequences of bovine *A3* genes. Through molecular phylogenetic analyses, we revealed that bovine *A3* genes, particularly *A3Z3*, are under strong positive selection. In addition, experimental analyses revealed that gaur A3Z3 is naturally resistant to degradation mediated by JDV Vif. Interestingly, the resistance of gaur A3Z3 to JDV Vif-mediated degradation was associated with the two amino acids positioned at 62 and 92, and both of these residues have been positively selected. Given that mammalian *A3* genes are assumed to have co-evolved with lentiviruses^9^,^11^,^12^,^37^,^43^, our findings suggest that gaur A3Z3 acquired resistance to JDV Vif-mediated degradation through selective pressure triggered by JDV or JDV-like viruses, which are extinct or have not yet been discovered. This finding is reminiscent of the observations of primate A3 and primate lentiviral Vif in which the host-virus interface between Vif and A3 suggests that lentiviruses can shape the evolution of the hosts that they infect^11^,^43^. Our findings, when taken together with these prior studies, support the concept that lentivirus-driven host evolution has occurred not only in primates but also in other mammals, including bovids.

**Figure 6:**
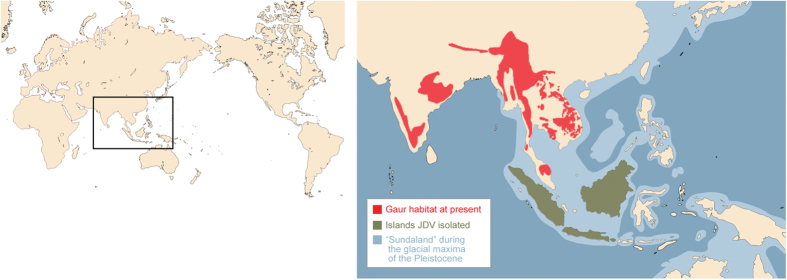
Biogeographical insight into the acquisition of resistance to JDV Vif-mediated degradation in gaur A3Z3. The Asian area boxed in the world map (left) is enlarged to the right. (Right) The land at present is shown in light beige. The current habitat of gaur (red) as stated by the website of the IUCN Red List of Threatened Species (http://www.iucnredlist.org/details/2891/0). The islands in Southeastern Asia, where JDV is detected (Bali, Borneo, Java, and Sumatra), is shown in green^18^,^20^,^44^,^45^. “Sundaland”, where gaur may have been exposed during the glacial maxima of the Pleistocene^46^, is shown in light blue. This image was created using Illustrator (Adobe) by overlaying the maps from the references above.

Our findings also suggest that gaur A3Z3 has been positively selected for escape from an ancestral JDV or JDV-like virus. However, gaur presently reside in mainland South/Southeast Asia, while JDV has been detected only on specific islands (Bali, Borneo, Java, and Sumatra) in Southeast Asia (Fig. 6)^18^,^20^,^44^,^45^. These insights indicate that JDV is geographically isolated from gaur and could not be the selective pressure for gaur A3Z3. However, geological studies have revealed that some Indonesian islands were connected with the Malay Peninsula on the Asian continent during the last glacial period (also known as the ice age; ca. 110,000 to 12,000 years ago), and this area is known as ‘Sundaland’ (Fig. 6)^46^. Importantly, all of the islands where JDV has been detected (Bali, Borneo, Java, and Sumatra islands) are part of Sundaland. Therefore, it would be plausible to assume that gaur were infected with JDV (or a JDV-like virus) in these areas in the past. Moreover, gaur has diversified from other bovids approximately 2.6 million years ago (Mya) (Fig. 1A)^14^. Taken together, our results suggest that gaur A3Z3 has been subjected to positive selective pressure from ancestral JDV acquired 2.6 Mya in Southeastern Asia, including the areas once known as Sundaland. It is also possible that gaur A3Z3 may have been positively selected by pressure exerted by extinct or presently undiscovered lentiviruses.

It should be noted that polymorphisms of *A3* genes have been described in certain mammals. For instance, interspecies variability of the *A3G* gene has been detected in certain Old World monkeys^11^,^12^, and it is known that some *A3* genes, particularly *A3D, A3F,* and *A3G*, are polymorphic in great apes^43^. In addition, *A3Z3,* which is also designated as *A3H* in primates, is polymorphic in humans (*Homo sapiens*)^47^,^48^ and domestic cat (*Felis catus*)^37^,^49^. In fact, here, we detected polymorphisms of the *A3Z1* and *A3Z3* genes in banteng (Fig. 1B). This finding raises the possibility of interspecies variability of *A3* genes in bovids, including gaur. However, in this study, we could only obtain one gaur sample (a gaur living in a Japanese zoo) for *A3* gene sequencing analyses. Gaur is an endangered species, and the import of livestock products is strictly prohibited in Japan. Access to a single sample makes it technically impossible to analyze the possibility of an *A3* polymorphism in gaur. Therefore, this issue will be addressed in a future study.

It should also be noted that only one strains of BIV (strain R29) and one strain of JDV (strain Tabanan/87) were used in this study because additional sequence information is not available in public databases. To fully elucidate the evolutionary relationship between bovids and their lentiviruses, further information on the sequences of bovine lentiviruses will be needed.

Previous studies on biogeography and molecular phylogenetics suggested that the genus *Bubalus*, including water buffalo (*Bubalus bubalis*), diversified from the genera *Bos* and *Bison* approximately 13.7 Mya^13^,^14^. Based on paleontological investigation, a fossil of the first animal in the tribe *Bovini* (*Selenoportax vexillarius*) was discovered in “Siwalik formation”, estimating it to be from approximately 11.2–19 Mya, in continental Southeastern Asia^15^. Because the *Bovini* tribe is monophyletic^14^, this paleontological and biogeographical evidence suggests that this tribe originated in Asia. Because JDV is an endemic lentivirus in Southeastern Asia, our results provide the first evidence, suggesting that a co-evolutionary arms race of mammals (bovids) and lentiviruses (JDV) occurred in Asia.

Here, we also demonstrated that bovine *A3Z3* is under strong positive selection, and the three amino acids positioned at 32, 62, and 92 are positively selected (Fig. 1C). In addition, two out of the three residues (those at positions 62 and 92) are closely associated with resistance to JDV Vif-mediated degradation of gaur A3Z3 protein (Fig. 5). Given that these two residues are likely exposed on the surface of the protein (Fig. 2), these residues in the bovine A3Z3 protein may interact with bovine lentiviral Vif proteins.

Interestingly, molecular phylogenetic analyses revealed that gaur A3Z3 has acquired an L62P substitution during its evolution (Fig. 5A), and protein homology modeling further suggested that the L62P substitution dramatically changes the conformation of the L1 domain, which is proximal to the amino acid at position 62 (Fig. 5D,E). To the best of our knowledge, our findings provide the first evidence that an amino acid change in the A3 protein indirectly affects its sensitivity to lentiviral Vif-mediated degradation by converting the structure of proximal residues.

## Methods

### Ethics statement

To determine the sequence of bovine *A3* and *CYTB*, fresh blood (cattle [*Bos taurus*; n = 2]), cryopreserved blood (zebu [*Bos indicus*; n = 1] and banteng [*Bos javanicus*; n = 3]), cryopreserved liver tissue (gaur [*Bos gaurus*; n = 1]), cryopreserved muscle tissue (European bison [*Bison bonasus*; n = 1]), or body hair root (yak [*Bos grunniens*; n = 1], American bison [*Bison bison*; n = 1], and water buffalo [*Bubalus bubalis*; n = 1]) were kindly provided by the following facilities: cattle blood from Tokyo University of Agriculture, Japan; zebu blood from the Miyazaki City Phoenix Zoo, Miyazaki, Japan; gaur liver from the Kanazawa Zoo, Kanagawa, Japan; European bison muscle from the Kyoto City Zoo, Kyoto, Japan; yak body hair from the Horii Zoo, Shiga, Japan; and body hairs of American bison and water buffalo from the Tobe Zoo, Ehime, Japan. Banteng blood was kindly provided by Dr. Takayuki Miyazawa (Institute for Virus Research, Kyoto University, Japan). Fresh cattle blood was collected in accordance with the guideline of Tokyo University of Agriculture, Japan. All experimental protocols were approved by a committee at Tokyo University of Agriculture, Japan.

### Sequencing PCR

Genomic DNA was extracted from blood and tissue samples (described above) using a DNeasy kit (Qiagen). In addition, genomic DNA was extracted from body hair root using a DNA Extractor FM kit (Wako). PCR was performed using PrimeSTAR GXL DNA polymerase (Takara); the primers are listed in Supplementary Table 4 (also illustrated in Supplementary Figure 1). The obtained PCR products were purified by gel extraction and then cloned using a Zero Blunt TOPO TA PCR Cloning Kit (Life Technologies). The nucleotide sequences were determined by a DNA sequencing service (Fasmac, Kanagawa, Japan), and the data were analyzed by Sequencher v5.1 software (Gene Codes Corporation).

### Molecular phylogenetic analyses

Molecular phylogenetic analyses were performed as previously described^37^,^50^,^51^. Briefly, the sequences of bovine *CYTB,* which were identified from the samples used in this study (Fig. 1A), were aligned with four *CYTB* sequences of respective species deposited in the GenBank database using ClustalW implemented in MEGA6^52^. The sequences of bovine *A3Z1, A3Z2,* and *A3Z3*, which were newly identified in this study (Fig. 1B and Table 1), were also aligned using ClustalW. The alignments were verified manually at the amino acid level. To select a proper evolutionary model, we used FindModel, which is a tool to select the best-fit model of nucleotide substitution^53^, and the GTR + G + I (general time-reversible model with gamma-distributed rate variation across sites and a proportion of invariable sites) was found to be the best model for the data. The phylogenetic trees were reconstructed using the maximum likelihood (ML) method with PhyML^54^, and the resulting trees were used for detecting positive selection (Fig. 1B). Because we were interested in whether *A3Z1, A3Z2* and *A3Z3* had been evolving under different selection pressures, two pairs of site models implemented in the PAML package v 4.7^55^ were employed to conduct the likelihood ratio tests for *A3Z1*, *A3Z2* and *A3Z3*, respectively (Fig. 1C and Supplementary Tables 1–3): M1 (neutral) versus M2 (selective) and M7 (neutral with beta) versus M8 (selective with beta). The sequences of ancestral bovine *A3Z3*, *Bos/Bison* and BCGZ MRCAs (Fig. 5) were inferred by using the site models as well (the nucleotide sequences are available upon request).

### Protein homology modeling

The 3D structure of bovine A3Z3 (Figs 2 and 5D,E) was simulated by the Swiss-Model server (http://swissmodel.expasy.org/) using the crystal structure of human A3F (PDB code: 4J4J) as a template^35^. The constructed structure model was then refined by energy minimization using the ModRefiner algorithm^56^. In Fig. 5E, the root mean square deviation (RMSD) value for Cα of the respective amino acid residue was calculated by comparing BCGZ MRCA A3Z3 to gaur A3Z3.

### Plasmid construction

To construct an HA-tagged cattle A3Z3 expression plasmid, RNA was extracted from cattle blood using an RNeasy Mini Kit (Qiagen). Reverse transcription was performed by the ThermoScript RT-PCR System (Life Technologies), and RT-PCR was performed using Platinum *Taq* DNA polymerase High Fidelity (Life Technologies) and the primers listed in Supplementary Table 5. To construct a series of bovine A3Z3 expression plasmids, overlap extension PCR was performed using the TOPO vectors cloned with each genomic region encoding an exon (see above and Supplementary Figure 1); the primers are listed in Supplementary Table 5. A series of bovine A3Z3 mutants (Fig. 5B,C) were prepared using a GeneArt site-directed mutagenesis system (Life Technologies), along with the primers listed in Supplementary Table 6. The flag-tagged codon-optimized open reading frames (ORFs) of BIV Vif (strain R29; accession no. M32690) and JDV Vif (strain Tabanan/87; U21603) were obtained from GeneArt Gene Synthesis service (Life Technologies; sequences are available upon request). The resultant DNA fragment was digested with BamHI and SalI and inserted into the BamHI-SalI site of the pDon-AI plasmid (Takara). A series of BIV/JDV Vif derivatives (Fig. 4B,D) were prepared using a Gibson assembly cloning kit (New England Biolabs) and the primers listed in Supplementary Table 7.

### Cell culture and transfection

HEK293T cells and TZM-bl cells (obtained through NIH AIDS Research and Reference Reagent Program) were maintained in Dulbecco’s modified Eagle medium (Sigma) containing 10% heat-inactivated fetal calf serum and antibiotics. Transfection was performed by using PEI Max (GE Healthcare) according to the manufacturer’s protocol. At 48 hours post-transfection, the culture supernatants and transfected cells were harvested and were respectively used for TZM-bl assay and Western blotting as described below.

### Western blotting and TZM-bl assay

The culture supernatant harvested at 48 hours post-transfection was centrifuged and filtered through a 0.45-μm filter (Millipore) to produce virus suspensions. The infectivity of the virus suspensions was measured by a TZM-bl assay as previously described^50^,^51^. Briefly, 100 μl of the virus solution were inoculated onto TZM-bl cells in a 96-well plate (Nunc), and the β-galactosidase activity was measured using the Galacto-Star mammalian reporter gene assay system (Applied Biosystems) and a 2030 ARVO X multilabel counter instrument (PerkinElmer) according to the manufacturers’ instructions. Western blotting was performed as previously described^37^,^50^,^51^ using the following antibodies: anti-CA polyclonal antibody (ViroStat), anti-flag polyclonal antibody (OctA; Santa Cruz Biotechnology), anti-HA antibody (3F10; Roche), and anti-alpha-Tubulin (TUBA) antibody (DM1A; Sigma). The band intensity of bovine A3Z3-HA (Figs 3A,C, 4B,D and 5B,C and Supplementary Figure 5) was quantified using ImageJ software (http://rsbweb.nih.gov/ij/).

### Statistical analyses

The data expressed as average with standard deviation (Fig. 3B,D), and significant differences were determined by Student’s *t* test.

### Nucleotide sequence accession number

The sequence of bovine *A3Z1, A3Z2,* and *A3Z3* have been submitted to DDBJ (accession numbers LC131290 to LC131328).

## Additional Information

**How to cite this article**: Yamada, E. *et al*. A naturally occurring bovine APOBEC3 confers resistance to bovine lentiviruses: implication for the co-evolution of bovids and their lentiviruses. *Sci. Rep.* **6**, 33988; doi: 10.1038/srep33988 (2016).

## Supplementary Material

Supplementary Information

## Figures and Tables

**Table 1:** Accession numbers of bovine *CYTB* and *APOBEC3* genes newly determined in this study.

Common name^a^	Scientific name	*Gene*	Accession no.^b^
Cattle	*Bos taurus*	*CYTB*	LC131290
Zebu	*Bos indicus*	*CYTB*	LC131291
Banteng #1, 3	*Bos javanicus*	*CYTB*	LC131292
Banteng #2	*Bos javanicus*	*CYTB*	LC131293
Gaur	*Bos gaurus*	*CYTB*	LC131294
European bison	*Bison bonasus*	*CYTB*	LC131295
American bison	*Bison bison*	*CYTB*	LC131296
Yak	*Bos grunniens*	*CYTB*	LC131297
Water buffalo	*Bubalus bubalis*	*CYTB*	LC131298
Cattle	*Bos taurus*	*APOBEC3Z1*	LC131299
Zebu	*Bos indicus*	*APOBEC3Z1*	LC131300
Banteng #1	*Bos javanicus*	*APOBEC3Z1*	LC131301
Banteng #2	*Bos javanicus*	*APOBEC3Z1*	LC131302
Banteng #3	*Bos javanicus*	*APOBEC3Z1*	LC131303
Gaur	*Bos gaurus*	*APOBEC3Z1*	LC131304
European bison	*Bison bonasus*	*APOBEC3Z1*	LC131305
American bison	*Bison bison*	*APOBEC3Z1*	LC131306
Yak	*Bos grunniens*	*APOBEC3Z1*	LC131307
Water buffalo	*Bubalus bubalis*	*APOBEC3Z1*	LC131308
Cattle	*Bos taurus*	*APOBEC3Z2*	LC131309
Zebu	*Bos indicus*	*APOBEC3Z2*	LC131310
Banteng #1	*Bos javanicus*	*APOBEC3Z2*	LC131311
Banteng #2	*Bos javanicus*	*APOBEC3Z2*	LC131312
Banteng #3	*Bos javanicus*	*APOBEC3Z2*	LC131313
Gaur	*Bos gaurus*	*APOBEC3Z2*	LC131314
European bison	*Bison bonasus*	*APOBEC3Z2*	LC131315
American bison	*Bison bison*	*APOBEC3Z2*	LC131316
Yak	*Bos grunniens*	*APOBEC3Z2*	LC131317
Water buffalo	*Bubalus bubalis*	*APOBEC3Z2*	LC131318
Cattle	*Bos taurus*	*APOBEC3Z3*	LC131319
Zebu	*Bos indicus*	*APOBEC3Z3*	LC131320
Banteng #1	*Bos javanicus*	*APOBEC3Z3*	LC131321
Banteng #2	*Bos javanicus*	*APOBEC3Z3*	LC131322
Banteng #3	*Bos javanicus*	*APOBEC3Z3*	LC131323
Gaur	*Bos gaurus*	*APOBEC3Z3*	LC131324
European bison	*Bison bonasus*	*APOBEC3Z3*	LC131325
American bison	*Bison bison*	*APOBEC3Z3*	LC131326
Yak	*Bos grunniens*	*APOBEC3Z3*	LC131327
Water buffalo	*Bubalus bubalis*	*APOBEC3Z3*	LC131328

^a^The common name of each bovid is identical to that in Fig. 1.

^b^The GenBank accession number (http://www.ncbi.nlm.nih.gov/genbank/) of each gene is listed.
