# Supplementary material for: A naturally occurring bovine APOBEC3 confers resistance to bovine lentiviruses: implication for the co-evolution of bovids and their lentiviruses
Source: Sci Rep. 2016 Sep 26;6:33988. doi: 10.1038/srep33988 (PMC5036201; doi:10.1038/srep33988)
Supplement: Supplementary Information [file srep33988-s1.pdf]

A naturally occurring bovine APOBEC3 confers resistance to bovine lentiviruses: implication for the co-evolution of bovids and their lentiviruses

Eri Yamada, Rokusuke Yoshikawa, Yusuke Nakano, Naoko Misawa, Tomoko Kobayashi, Fengrong Ren, Taisuke Izumi, Takayuki Miyazawa, Yoshio Koyanagi, and Kei Sato.

Supplementary Information:

Supplementary figure 1: Scheme of bovine genome encoding *APOBEC3* and the position of the primers used in this study
Supplementary figure 2: Summary of putative bovine ISRE
Supplementary figure 3: Unrooted phylogenetic tree of all bovine *A3* genes
Supplementary figure 4: Identity and similarity of BIV Vif and JDV Vif
Supplementary figure 5: Degradation of bovine APOBEC3Z2Z3 by BIV Vif and JDV Vif
Supplementary figures 6-9: Original (uncropped) blots
Supplementary tables 1-3: Summary of site model analyses on bovine APOBEC3Z1-Z3
Supplementary table 4: Primers used for bovine APOBEC3 and CYTB sequencing
Supplementary table 5: Primers used for the construction of bovine APOBEC3Z3 expression plasmids
Supplementary table 6: Primers used for the construction of bovine APOBEC3 MRCA expression plasmids
Supplementary table 7: Primers used for the construction of expression plasmids for BIV/JDV Vif derivatives

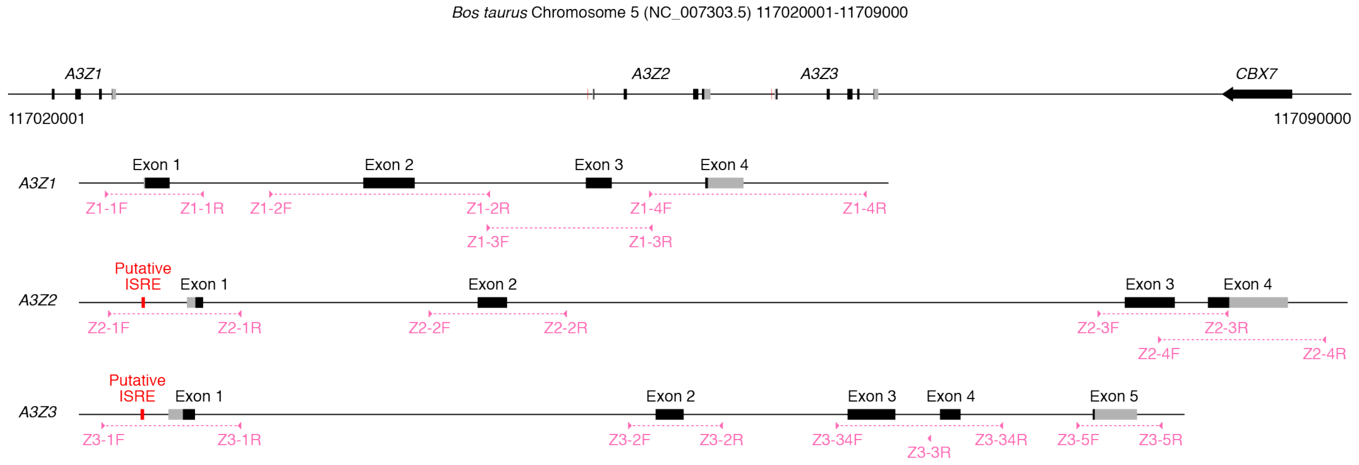

Supplementary Figure 1. Scheme of bovine genome encoding *APOBEC3* and the position of the primers used in this study – Related to Figure 1.
The scheme of *Bos taurus* chromosome 5 including the exons of bovine *APOBEC3Z1, Z2,* and *Z3* is shown. The primers used for PCR/sequencing are shown in pink arrowheads, and the names are identical to those in **Supplementary table 4**.

	A3Z2 putative ISRE	*A3Z3* putative ISRE
Cattle	TTTACTTTCTCTTTCCCTTT	TTTACTTTCTCTTTCCCCTT
Zebu	....	....
European bison	....	....
Banteng	....	....
Gaur	....	....
American bison	....	....
Yak	....	....
Water buffalo	....	..........G.........

Supplementary Figure 2. Summary of putative bovine ISRE.
The sequence of putative ISRE of each bovid, which is indicated by red in **Supplementary figure 1**, is summarized.

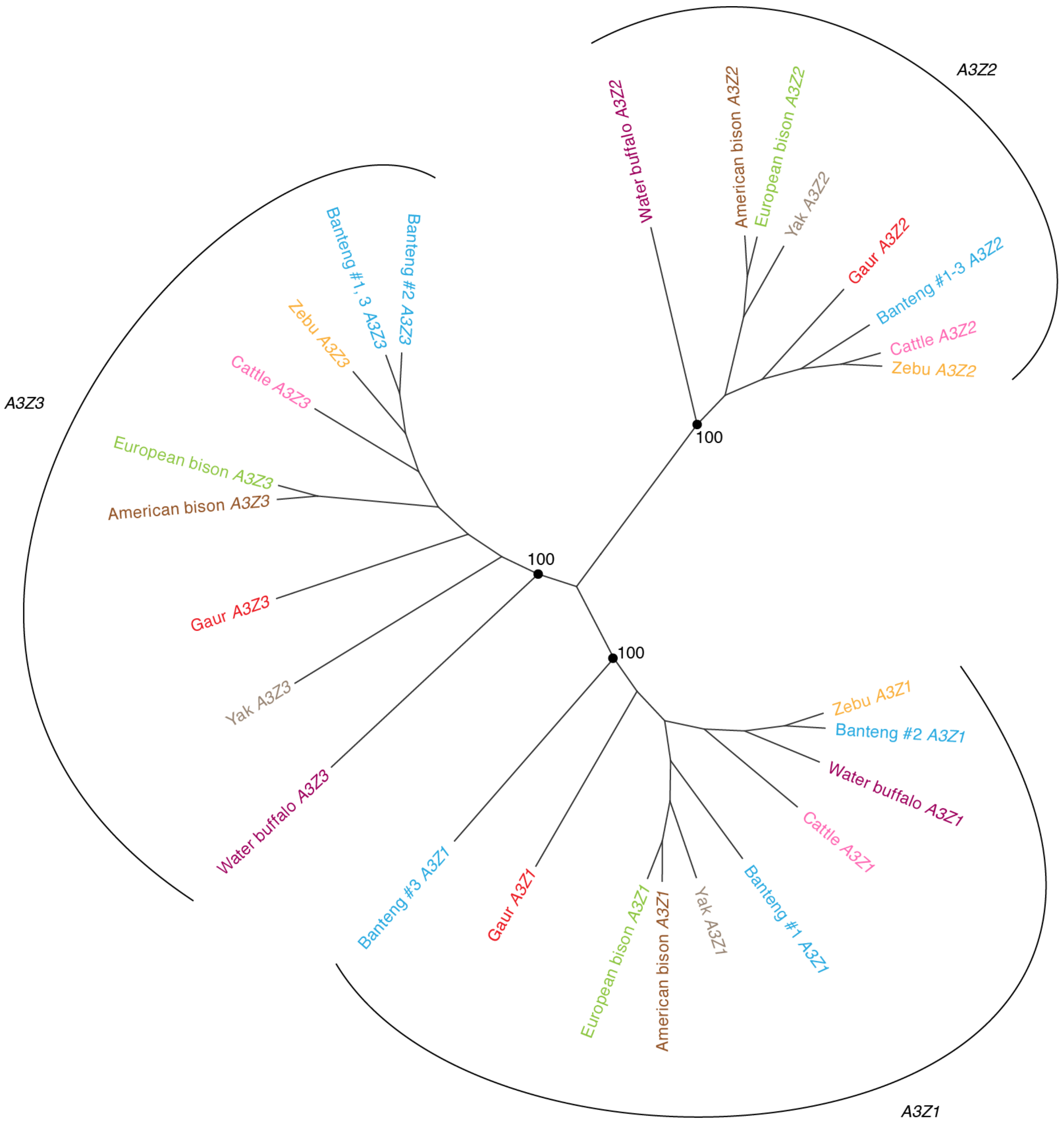

Supplementary Figure 3. Unrooted phylogenetic tree of all bovine *A3* genes. Phylogenetic tree of all bovine *A3* genes reconstructed using ML method. The bootstrap value for each cluster (indicated with black dot) is indicated.

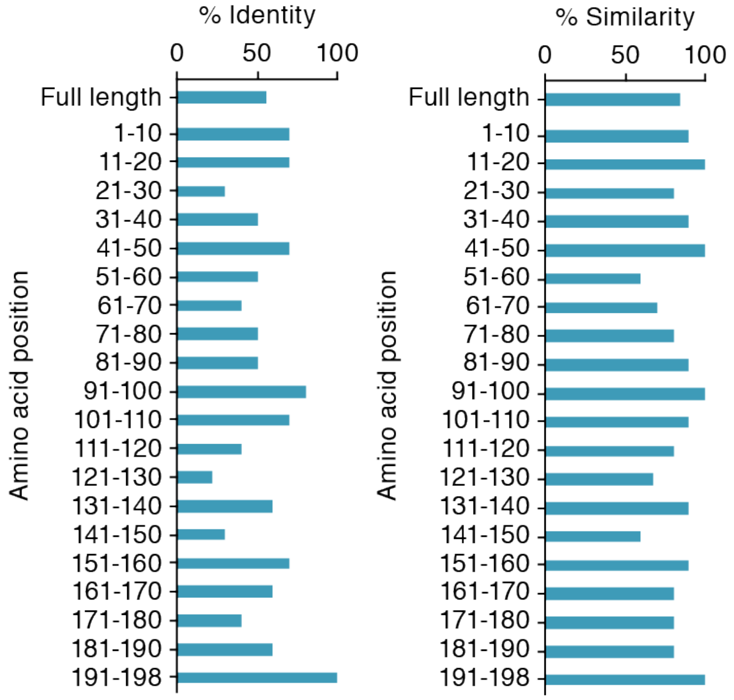

Supplementary Figure 4. Identity and similarity of BIV Vif and JDV Vif – Related to Figure 4.
The identity (left) and similarity (right) of BIV/JDV Vif is summarized.

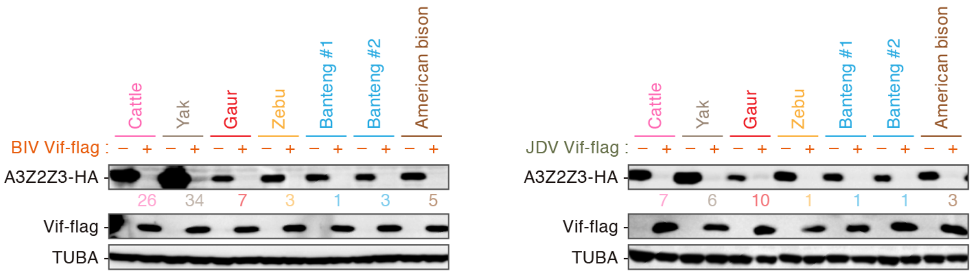

Supplementary Figure 5. Degradation of bovine APOBEC3Z2Z3 by BIV Vif and JDV Vif.

The HA-tagged expression plasmids for bovine APOBEC3Z2Z3 (150 ng) was co-transfected with the flag-tagged expression plasmid for either BIV Vif or JDV Vif (1,000 ng). Representative results of Western blotting are shown. The value under the band of respective bovine A3Z2Z3-HA represents the percentage of band intensity compared to the absence of Vif. Blots have been cropped; full uncropped blots are available as **Supplementary figure 7**.

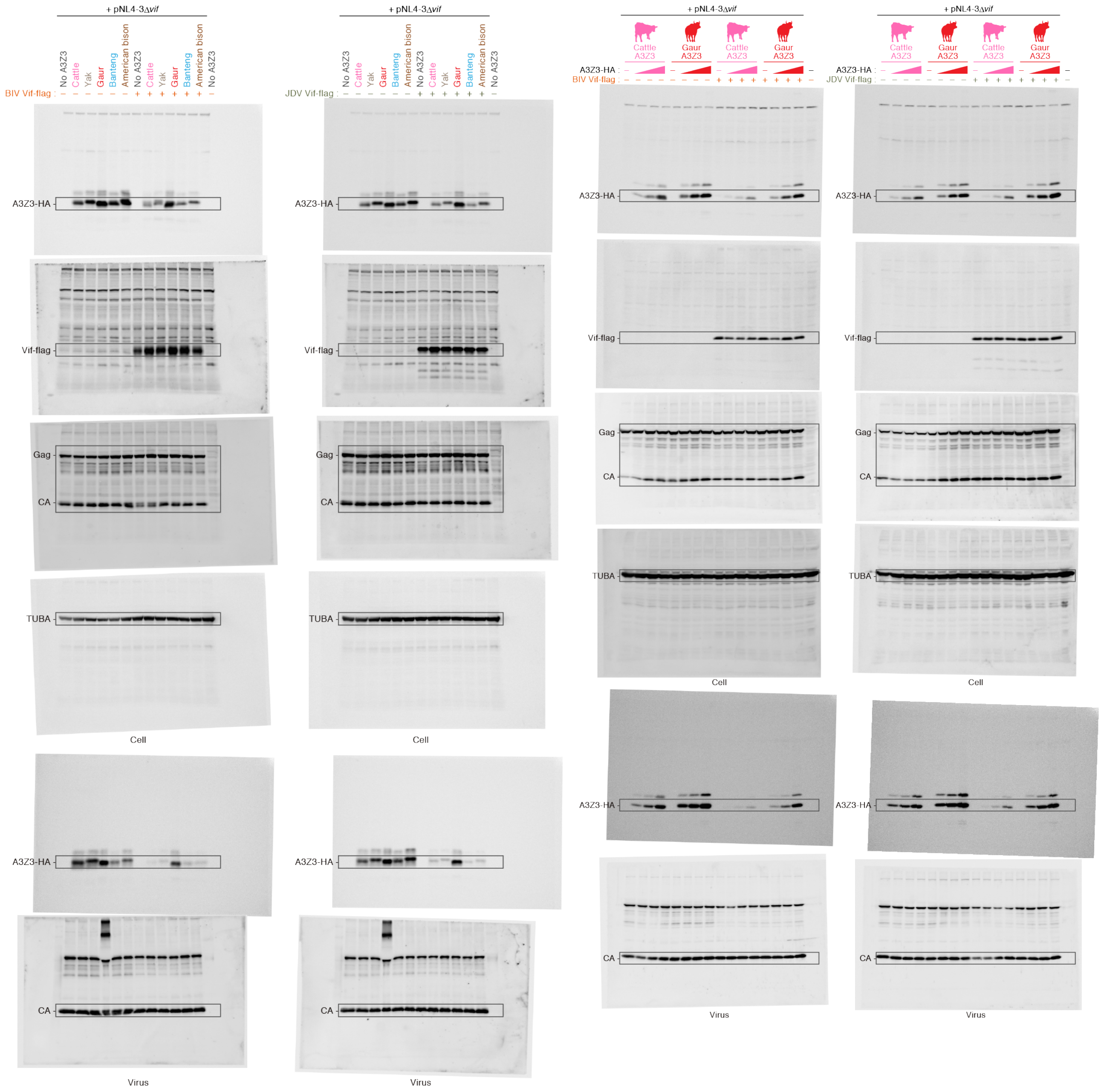

Supplementary Figure 6. Original (uncropped) blots of Figures 3A and 3C.

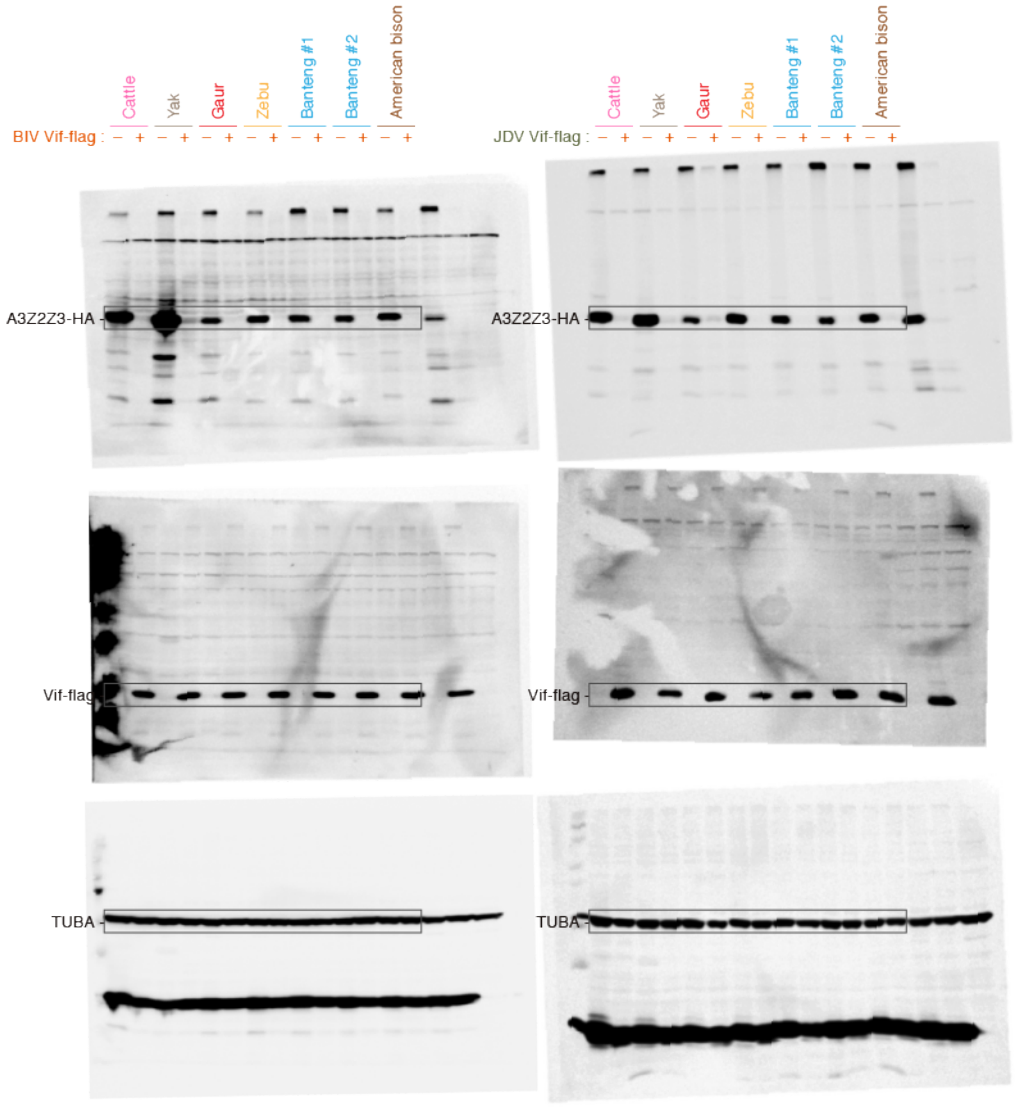

Supplementary Figure 7. Original (uncropped) blots of Supplementary figure 5.

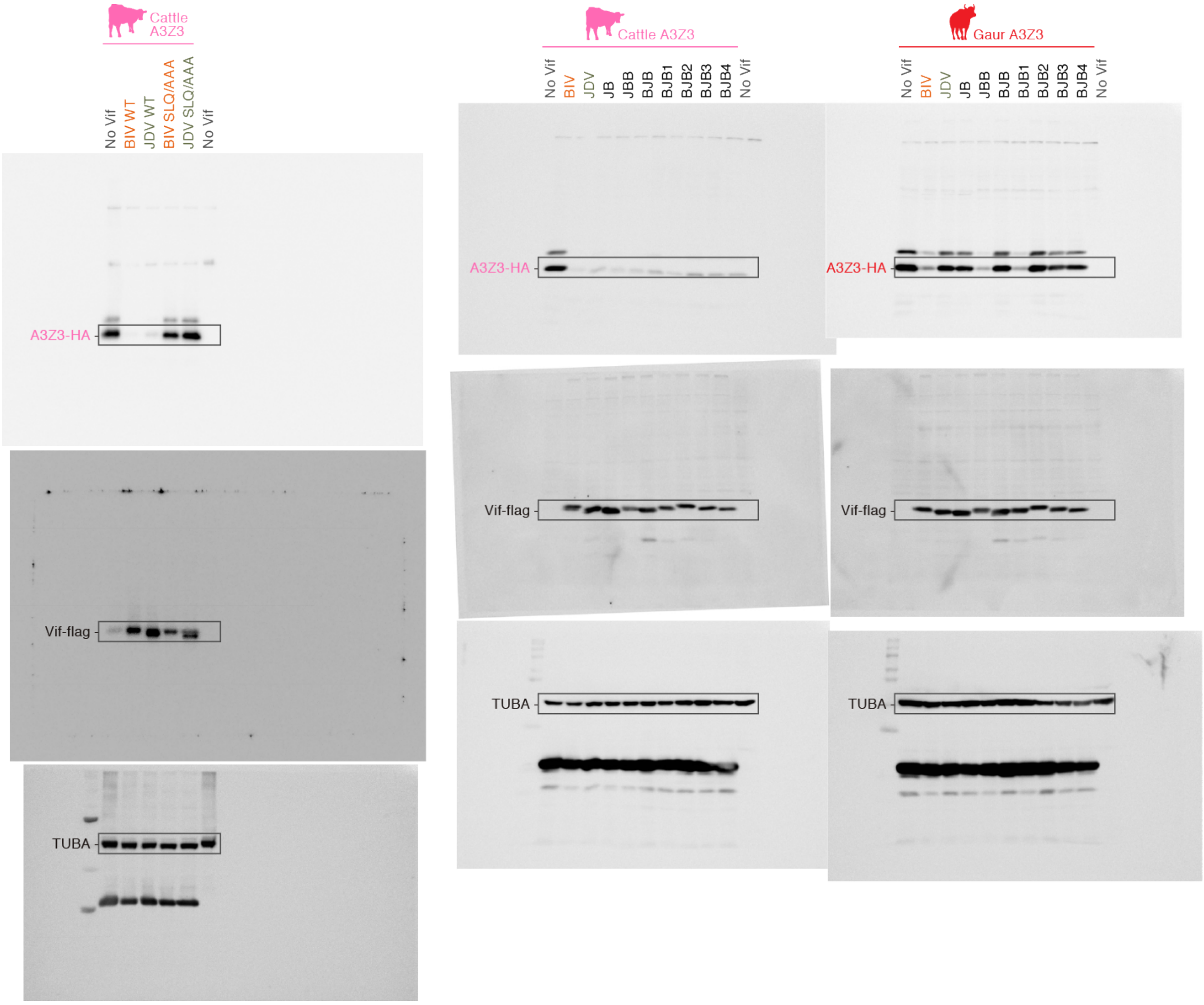

Supplementary Figure 8. Original (uncropped) blots of Figures 4B and 4D.

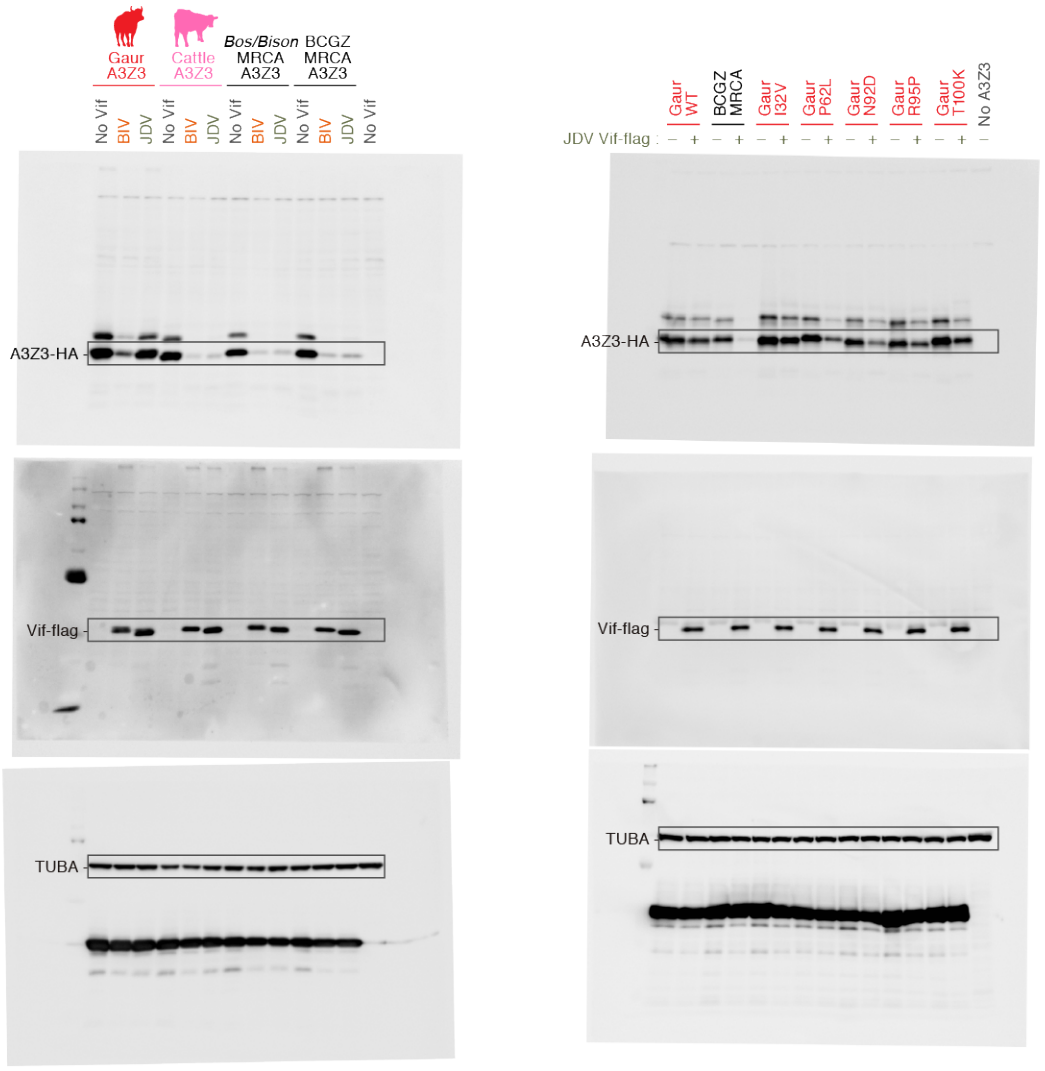

Supplementary Figure 9. Original (uncropped) blots of Figures 5B and 5C.

Supplementary table 1. Summary of site model analyses on bovine *APOBEC3Z1*.

Site	Posterior probability	dN/dS ratio
1	0.19	1.40
2	0.18	1.28
3	0.22	1.60
4	0.21	1.54
5	0.19	1.41
6	0.20	1.42
7	0.22	1.59
8	0.19	1.36
9	0.18	1.29
10	0.20	1.42
11	0.18	1.29
12	0.21	1.51
13	0.23	1.68
14	0.77	5.43
15	0.96	6.72
16	0.22	1.61
17	0.74	5.22
18	0.19	1.37
19	0.19	1.41
20	0.18	1.32
21	0.22	1.61
22	0.19	1.35
23	0.18	1.32
24	0.19	1.36
25	0.74	5.20
26	0.19	1.36
27	0.19	1.37
28	0.22	1.61
29	0.18	1.28
30	0.20	1.43
31	0.69	4.88
32	0.22	1.60
33	0.20	1.47
34	0.19	1.40
35	0.22	1.61
36	0.22	1.61
37	0.18	1.28
38	0.19	1.36
39	0.18	1.32
40	0.19	1.37
41	0.18	1.32
42	0.22	1.60
43	0.20	1.47
44	0.18	1.33
45	0.18	1.29
46	0.19	1.37
47	0.21	1.54
48	0.23	1.65
49	0.75	5.27
50	0.23	1.68
51	0.21	1.51
52	0.19	1.36
53	0.96	6.72
54	0.74	5.22
55	0.19	1.35
56	0.21	1.53
57	0.23	1.67
58	0.19	1.36
59	0.20	1.44
60	0.18	1.32
61	0.20	1.42
62	0.22	1.61
63	0.23	1.64
64	0.19	1.37
65	0.19	1.40
66	0.75	5.32
67	0.22	1.62
68	0.22	1.60
69	0.21	1.51
70	0.22	1.61
71	0.18	1.28
72	0.20	1.46
73	0.21	1.51
74	0.22	1.56
75	0.18	1.33
76	0.18	1.32
77	0.19	1.37
78	0.20	1.44
79	0.19	1.41
80	0.22	1.56
81	0.20	1.42
82	0.19	1.40
83	0.20	1.45
84	0.22	1.60
85	0.18	1.29
86	0.20	1.43
87	0.22	1.56
88	0.18	1.32
89	0.21	1.50
90	0.22	1.56
91	0.20	1.41
92	0.22	1.56
93	0.22	1.60
94	0.22	1.61
95	0.22	1.59
96	0.23	1.67
97	0.20	1.42
98	0.23	1.63
99	0.19	1.37
100	0.19	1.36
101	0.18	1.29
102	0.71	4.97
103	0.18	1.33
104	0.21	1.50
105	0.18	1.29
106	0.22	1.61
107	0.18	1.33
108	0.19	1.40
109	0.22	1.61
110	0.20	1.41
111	0.20	1.45
112	0.18	1.33
113	0.19	1.40
114	0.21	1.54
115	0.19	1.41
116	0.95	6.64
117	0.70	4.94
118	0.75	5.33
119	0.22	1.60
120	0.19	1.39
121	0.19	1.35
122	0.21	1.53
123	0.22	1.56
124	0.21	1.52
125	0.18	1.32
126	0.22	1.61
127	0.19	1.35
128	0.22	1.60
129	0.22	1.61
130	0.23	1.68
131	0.20	1.48
132	0.22	1.60
133	0.19	1.39
134	0.20	1.41
135	0.23	1.68
136	0.19	1.40
137	0.19	1.41
138	0.19	1.40
139	0.19	1.40
140	0.19	1.41
141	0.22	1.60
142	0.19	1.36
143	0.18	1.28
144	0.22	1.60
145	0.19	1.37
146	0.18	1.33
147	0.19	1.35
148	0.22	1.60
149	0.22	1.60
150	0.19	1.41
151	0.20	1.42
152	0.20	1.47
153	0.18	1.28
154	0.18	1.33
155	0.19	1.37
156	0.23	1.64
157	0.22	1.60
158	0.20	1.43
159	0.22	1.60
160	0.22	1.56
161	0.20	1.43
162	0.22	1.60
163	0.19	1.36
164	0.19	1.39
165	0.24	1.73
166	0.21	1.51
167	0.23	1.67
168	0.19	1.37
169	0.21	1.52
170	0.22	1.56
171	0.21	1.50
172	0.20	1.44
173	0.22	1.56
174	0.75	5.27
175	0.22	1.60
176	0.22	1.61
177	0.22	1.56
178	0.20	1.41
179	0.22	1.58
180	0.20	1.44
181	0.19	1.37
182	0.22	1.59
183	0.22	1.56
184	0.23	1.68
185	0.18	1.29

Supplementary table 2. Summary of site model analyses on bovine *APOBEC3Z2*.

Site	Posterior probability	dN/dS ratio	Site	Posterior probability	dN/dS ratio	Site	Posterior probability	dN/dS ratio	Site	Posterior probability	dN/dS ratio
1	0.30	1.74	65	0.30	1.78	129	0.29	1.68	193	0.32	1.87
2	0.32	1.89	66	0.30	1.76	130	0.30	1.73	194	0.33	1.92
3	0.33	1.93	67	0.32	1.88	131	0.30	1.73	195	0.32	1.87
4	0.31	1.78	68	0.32	1.85	132	0.30	1.77	196	0.30	1.74
5	0.29	1.69	69	0.29	1.66	133	0.79	4.87	197	0.33	1.91
6	0.33	1.94	70	0.31	1.80	134	0.32	1.90	198	0.30	1.74
7	0.78	4.81	71	0.30	1.76	135	0.30	1.76	199	0.29	1.66
8	0.29	1.69	72	0.32	1.89	136	0.29	1.69	200	0.32	1.89
9	0.29	1.68	73	0.81	4.98	137	0.78	4.80	201	0.30	1.76
10	0.80	4.90	74	0.30	1.78	138	0.30	1.73	202	0.30	1.78
11	0.30	1.74	75	0.33	1.92	139	0.31	1.80			
12	0.30	1.77	76	0.78	4.81	140	0.30	1.74			
13	0.31	1.83	77	0.29	1.69	141	0.30	1.74			
14	0.30	1.77	78	0.29	1.66	142	0.96	5.92			
15	0.32	1.88	79	0.30	1.77	143	0.30	1.77			
16	0.29	1.66	80	0.32	1.87	144	0.30	1.75			
17	0.30	1.77	81	0.30	1.76	145	0.31	1.80			
18	0.29	1.68	82	0.29	1.71	146	0.30	1.78			
19	0.32	1.87	83	0.30	1.73	147	0.32	1.87			
20	0.29	1.66	84	0.32	1.87	148	0.30	1.76			
21	0.30	1.74	85	0.33	1.92	149	0.30	1.75			
22	0.33	1.93	86	0.31	1.83	150	0.32	1.87			
23	0.31	1.79	87	0.30	1.73	151	0.29	1.68			
24	0.33	1.94	88	0.77	4.73	152	0.29	1.66			
25	0.32	1.85	89	0.31	1.78	153	0.31	1.80			
26	0.33	1.92	90	0.30	1.74	154	0.33	1.92			
27	0.32	1.87	91	0.30	1.73	155	0.31	1.78			
28	0.30	1.77	92	0.32	1.87	156	0.32	1.85			
29	0.30	1.73	93	0.29	1.71	157	0.31	1.81			
30	0.29	1.69	94	0.81	5.00	158	0.29	1.66			
31	0.30	1.73	95	0.29	1.66	159	0.32	1.89			
32	0.31	1.80	96	0.30	1.74	160	0.30	1.74			
33	0.32	1.88	97	0.32	1.90	161	0.30	1.77			
34	0.96	5.91	98	0.29	1.69	162	0.29	1.69			
35	0.29	1.66	99	0.29	1.66	163	0.32	1.89			
36	0.33	1.95	100	0.30	1.73	164	0.32	1.87			
37	0.30	1.76	101	0.30	1.77	165	0.30	1.73			
38	0.32	1.89	102	0.31	1.83	166	0.32	1.86			
39	0.31	1.78	103	0.30	1.73	167	0.29	1.69			
40	0.29	1.66	104	0.30	1.74	168	0.32	1.87			
41	0.30	1.75	105	0.33	1.95	169	0.31	1.83			
42	0.30	1.75	106	0.31	1.83	170	0.32	1.86			
43	0.29	1.66	107	0.29	1.68	171	0.30	1.73			
44	0.29	1.69	108	0.30	1.77	172	0.32	1.88			
45	0.79	4.87	109	0.29	1.69	173	0.33	1.92			
46	0.32	1.89	110	0.30	1.74	174	0.77	4.75			
47	0.30	1.73	111	0.30	1.73	175	0.32	1.86			
48	0.29	1.69	112	0.32	1.87	176	0.31	1.80			
49	0.29	1.69	113	0.31	1.83	177	0.31	1.81			
50	0.30	1.73	114	0.30	1.73	178	0.30	1.75			
51	0.31	1.81	115	0.30	1.74	179	0.30	1.77			
52	0.32	1.87	116	0.31	1.81	180	0.30	1.73			
53	0.32	1.88	117	0.33	1.92	181	0.30	1.75			
54	0.30	1.75	118	0.29	1.66	182	0.33	1.92			
55	0.32	1.86	119	0.30	1.73	183	0.31	1.83			
56	0.29	1.69	120	0.32	1.87	184	0.30	1.73			
57	0.29	1.66	121	0.30	1.78	185	0.32	1.86			
58	0.33	1.95	122	0.30	1.75	186	0.33	1.95			
59	0.32	1.90	123	0.29	1.66	187	0.29	1.69			
60	0.79	4.87	124	0.31	1.80	188	0.32	1.88			
61	0.30	1.77	125	0.31	1.84	189	0.31	1.84			
62	0.30	1.73	126	0.31	1.81	190	0.32	1.89			
63	0.29	1.66	127	0.31	1.82	191	0.31	1.80			
64	0.31	1.83	128	0.32	1.87	192	0.31	1.79			

Supplementary table 3. Summary of site model analyses on bovine *APOBEC3Z3*.

Site	Posterior probability	dN/dS ratio	Site	Posterior probability	dN/dS ratio	Site	Posterior probability	dN/dS ratio	Site	Posterior probability	dN/dS ratio
1	0.31	2.60	65	0.33	2.76	129	0.32	2.75	193	0.31	2.61
2	0.31	2.66	66	0.31	2.67	130	0.31	2.63	194	0.32	2.69
3	0.31	2.63	67	0.33	2.78	131	0.31	2.64	195	0.32	2.75
4	0.31	2.66	68	0.31	2.61	132	0.33	2.77	196	0.33	2.81
5	0.32	2.74	69	0.32	2.75	133	0.32	2.69	197	0.31	2.63
6	0.33	2.79	70	0.31	2.66	134	0.32	2.70	198	0.32	2.68
7	0.31	2.67	71	0.31	2.67	135	0.31	2.66	199	0.32	2.69
8	0.33	2.82	72	0.31	2.67	136	0.32	2.74	200	0.31	2.63
9	0.31	2.66	73	0.31	2.66	137	0.31	2.65	201	0.31	2.64
10	0.32	2.73	74	0.31	2.59	138	0.31	2.67	202	0.33	2.82
11	0.33	2.79	75	0.31	2.61	139	0.31	2.62	203	0.31	2.67
12	0.31	2.67	76	0.31	2.61	140	0.32	2.74	204	0.32	2.69
13	0.33	2.77	77	0.32	2.69	141	0.31	2.64	205	0.33	2.79
14	0.33	2.79	78	0.32	2.68	142	0.31	2.61	206	0.32	2.75
15	0.33	2.79	79	0.32	2.73	143	0.88	7.24			
16	0.31	2.67	80	0.33	2.80	144	0.33	2.77			
17	0.33	2.78	81	0.32	2.75	145	0.31	2.61			
18	0.32	2.74	82	0.32	2.75	146	0.31	2.60			
19	0.33	2.77	83	0.31	2.66	147	0.31	2.67			
20	0.32	2.69	84	0.33	2.77	148	0.32	2.74			
21	0.33	2.79	85	0.32	2.75	149	0.32	2.69			
22	0.31	2.66	86	0.31	2.61	150	0.32	2.73			
23	0.32	2.75	87	0.31	2.61	151	0.32	2.74			
24	0.31	2.59	88	0.31	2.64	152	0.32	2.69			
25	0.31	2.67	89	0.31	2.59	153	0.87	7.18			
26	0.31	2.60	90	0.33	2.82	154	0.33	2.79			
27	0.32	2.72	91	0.32	2.74	155	0.31	2.67			
28	0.32	2.74	92	0.99	8.10	156	0.31	2.64			
29	0.33	2.82	93	0.32	2.74	157	0.33	2.82			
30	0.32	2.75	94	0.31	2.59	158	0.32	2.67			
31	0.32	2.75	95	0.87	7.19	159	0.33	2.79			
32	0.99	8.10	96	0.31	2.61	160	0.31	2.67			
33	0.32	2.74	97	0.32	2.69	161	0.31	2.60			
34	0.31	2.67	98	0.31	2.61	162	0.31	2.66			
35	0.31	2.61	99	0.31	2.65	163	0.31	2.62			
36	0.32	2.69	100	0.87	7.21	164	0.33	2.78			
37	0.32	2.69	101	0.31	2.64	165	0.31	2.63			
38	0.33	2.77	102	0.31	2.64	166	0.33	2.77			
39	0.31	2.66	103	0.31	2.67	167	0.32	2.75			
40	0.31	2.59	104	0.33	2.77	168	0.31	2.61			
41	0.32	2.69	105	0.31	2.64	169	0.31	2.67			
42	0.32	2.69	106	0.33	2.78	170	0.32	2.74			
43	0.32	2.68	107	0.32	2.74	171	0.32	2.75			
44	0.31	2.67	108	0.31	2.61	172	0.32	2.69			
45	0.33	2.79	109	0.33	2.79	173	0.33	2.77			
46	0.33	2.80	110	0.31	2.67	174	0.32	2.67			
47	0.33	2.79	111	0.32	2.69	175	0.31	2.61			
48	0.31	2.65	112	0.31	2.59	176	0.31	2.59			
49	0.31	2.65	113	0.33	2.77	177	0.32	2.69			
50	0.32	2.68	114	0.32	2.69	178	0.33	2.82			
51	0.31	2.63	115	0.32	2.72	179	0.31	2.63			
52	0.31	2.61	116	0.31	2.63	180	0.32	2.69			
53	0.31	2.66	117	0.32	2.74	181	0.31	2.67			
54	0.31	2.65	118	0.31	2.67	182	0.32	2.69			
55	0.32	2.74	119	0.32	2.72	183	0.32	2.69			
56	0.31	2.67	120	0.31	2.67	184	0.32	2.74			
57	0.31	2.65	121	0.31	2.64	185	0.31	2.61			
58	0.32	2.69	122	0.31	2.66	186	0.31	2.61			
59	0.32	2.74	123	0.31	2.63	187	0.32	2.74			
60	0.31	2.61	124	0.32	2.72	188	0.31	2.63			
61	0.32	2.69	125	0.31	2.59	189	0.33	2.77			
62	0.99	8.10	126	0.31	2.62	190	0.31	2.65			
63	0.32	2.72	127	0.32	2.74	191	0.32	2.73			
64	0.31	2.61	128	0.32	2.73	192	0.32	2.69			

Supplementary table 4. Primers used for bovine *APOBEC3* and *CYTB* sequencing.[a]

Primer name	Sequences (5' to 3')	Product (bp)
Z1-1F	agaggactgggtcatcatcg	552
Z1-1R	tctgacatgtggctcaaagc	
Z1-2F	ggttagtggggtagggtcgt	1232
Z1-2R	acttcgtcagcacgttcctt	
Z1-3F	aaggaacgtgctgacgaagt	925
Z1-3R	tggtctgggaatgaagatcc	
Z1-4F	ggatcttcattcccagacca	1214
Z1-4R	tcctctccagggcagtagaa	
Z2-1F	cctacctcataagggccaca	744
Z2-1R	ggccagttcagaagcaagag	
Z2-2F	tggggattagggtgctacag	773
Z2-2R	agcaccgagtcttcaccact	
Z2-3F	agaaaagaagggaggggaca	730
Z2-3R	ccgagaatgtcctcaagctc	
Z2-4F	gcccgtctctacaacttcca	937
Z2-4R	gaaaggccatctgaggacac	
Z3-1F	cacagatctgcccaacacag	780
Z3-1R	actggcatcgatacctggtc	
Z3-2F	ctggtcagccaagaaggaag	528
Z3-2R	gagctgagacctggaggatg	
Z3-34F	atgtgtgacctctggcctct	934
Z3-34R	atcagggaagccacaaagaa	
Z3-5F	ctagtccctgcgttttcagc	479
Z3-5R	atggtcccactgttccagac	
Bos CYTB Fwd	acaaatcctcacaggcctattc	
Bos CYTB Rev	taggacgtatcctatgaatgct	

[a] The primer names are identical to those in Supplementary figure 1.

Supplementary table 5. Primers used for the construction of bovine APOBEC3Z3 expression plasmids.

Primer name	Sequences (5' to 3')	Template	Species	Purpose
btA3Z3-HA Fwd	tatatatatataaagcttgtcctgggagtgtctatga	cDNA	Cattle	HA-tagged ORF preparation
btA3Z3-HA Rev	ttttttttttctcgagtcaagcgtaatctggaacatcgtatgggtaaattggggccgttaggatc	cDNA	Cattle	HA-tagged ORF preparation
BGY Z3 exons 1,2 Fwd	aaacacgatgaatctgctaagagaaattctgttcaagcagcag	genomic DNA	Banteng, Gaur and Yak	ORF/exon conjugation
BGY Z3 exons 1,2 Rev	ctgcttgaacagaatttctcttagcagattcatcgtgtttctggttccgggagtccaaac	genomic DNA	Banteng, Gaur and Yak	ORF/exon conjugation
BGY Z3 exons 2,3 Fwd	gaggctgcttccgcaacaagaagcagcggcatgcagaaattc	genomic DNA	Banteng, Gaur and Yak	ORF/exon conjugation
BGY Z3 exons 2,3 Rev	atttctgcatgccgctgcttcttgttgcggaagcagcc	genomic DNA	Banteng, Gaur and Yak	ORF/exon conjugation
BGY Z3 exons 3,4 Fwd	ggctgtcatgacccacacagagtttgaagactgctgggaac	genomic DNA	Banteng, Gaur and Yak	ORF/exon conjugation
BGY Z3 exons 3,4 Rev	ttcccagcagtcttcaaactctgtgtgggtcatgacagcc	genomic DNA	Banteng, Gaur and Yak	ORF/exon conjugation
BGYA Z3 exons 4,5 Rev	aattggggccgttaggatcctctggagcc	genomic DNA	American bison, Banteng, Gaur and Yak	ORF/exon conjugation
American bison Z3 exons 1,2 Fwd	gactcccggaaccagaaacacgatgaatctgct	genomic DNA	American bison	ORF/exon conjugation
American bison Z3 exons 1,2 Rev	agattcatcgtgtttctggttccgggagtccaa	genomic DNA	American bison	ORF/exon conjugation
American bison Z3 exons 2,3 Fwd	gcttccgcaacaagaagcagcggcatgcagaaatt	genomic DNA	American bison	ORF/exon conjugation
American bison Z3 exons 2,3 Rev	tgcatgccgctgcttcttgttgcggaagcagcc	genomic DNA	American bison	ORF/exon conjugation
American bison Z3 exons 3,4 Fwd	tcatgacccacacagagtttgaagactgctgg	genomic DNA	American bison	ORF/exon conjugation
American bison Z3 exons 3,4 Rev	agcagtcttcaaactctgtgtgggtcatgacag	genomic DNA	American bison	ORF/exon conjugation

Supplementary table 6. Primers used for the construction of bovine APOBEC3 MRCA expression plasmids.

Primer name	Sequences (5' to 3')	Template	Product
Yak Z3 I32V Fwd	ctgctaagagaagttctgttcaagcagc	Yak APOBEC3Z3, Gaur APOBEC3Z3	*Bos/Bison* MRCA, Gaur APOBEC3Z3 V32I
Yak Z3 I32V Rev	gctgcttgaacagaacttctcttagcag	Yak APOBEC3Z3, Gaur APOBEC3Z3	*Bos/Bison* MRCA, Gaur APOBEC3Z3 V32I
BB MRCA P143S Fwd	ccactggatcaagtcatttaagatggggct	*Bos/Bison* MRCA	BCGZ MRCA
BB MRCA P143S Rev	agccccatcttaaatgacttgatccagtgg	*Bos/Bison* MRCA	BCGZ MRCA
Gaur APOBEC3Z3 P62L Fwd	cagctgaagcagcttaatgacttgacgc	Gaur APOBEC3Z3	Gaur APOBEC3Z3 P62L
Gaur APOBEC3Z3 P62L Rev	gcgtcaagtcattaagctgcttcagctg	Gaur APOBEC3Z3	Gaur APOBEC3Z3 P62L
Gaur APOBEC3Z3 N92D Fwd	gatcaactcactggatctgaaccgga	Gaur APOBEC3Z3	Gaur APOBEC3Z3 N92D
Gaur APOBEC3Z3 N92D Rev	tccggttcagatccagtgagttgatc	Gaur APOBEC3Z3	Gaur APOBEC3Z3 N92D
Gaur APOBEC3Z3 R95P Fwd	ctgaatctgaacccgagccagagctaca	Gaur APOBEC3Z3	Gaur APOBEC3Z3 R95P
Gaur APOBEC3Z3 R95P Rev	tgtagctctggctcgggttcagattcag	Gaur APOBEC3Z3	Gaur APOBEC3Z3 R95P
Gaur APOBEC3Z3 T100K Fwd	cggagccagagctacaaaatcatctgctatatcac	Gaur APOBEC3Z3	Gaur APOBEC3Z3 T100K
Gaur APOBEC3Z3 T100K Rev	gtgatatagcagatgattttgtagctctggctccg	Gaur APOBEC3Z3	Gaur APOBEC3Z3 T100K

Supplementary table 7. Primers used for the construction of expression plasmids for BIV/JDV Vif derivatives.

Primer name	Sequences (5' to 3')	Product
BIV Vif SLQ/AAA Fwd	gcacacctagacacgccgccgccagactggccgccctg	BIV Vif SLQ/AAA
BIV Vif SLQ/AAA Rev	cagggcggccagtctggcggcggcgtgtctaggtgtgc	BIV Vif SLQ/AAA
JDV Vif SLQ/AAA Fwd	cacaccaaacctcacgccgccgccagactggccggcctg	JDV Vif SLQ/AAA
JDV Vif SLQ/AAA Rev	caggccggccagtctggcggcggcgtgaggtttggtgtg	JDV Vif SLQ/AAA
Vif JB Fwd	gtcgacgttaacgcatgc	Vif JB
Vif JB Rev	ccagaacaggtccagctc	Vif JB
Vif JB in-Fwd	ctggacctgttctgggtgcgctacaccctgtgc	Vif JB
Vif JB in-Rev	atgcgttaacgtcgactcatttgtcgtcgtcgtcc	Vif JB
Vif JBB Fwd	gcggcagaccgagtacag	Vif JBB
Vif JBB Rev	agcttaagtttaaacgctagcgg	Vif JBB
Vif JBB in-Fwd	agcgtttaaacttaagcttggtaccgagctcggatc	Vif JBB
Vif JBB in-Rev	gtactcggtctgccgcacgaactcccatctggg	Vif JBB
Vif BJB Fwd	ctgcggtactgcagctgc	Vif BJB
Vif BJB Rev	cacgaactcccatctggg	Vif BJB
Vif BJB in-Fwd	cagatgggagttcgtgatgaacgacctgtacagc	Vif BJB
Vif BJB in-Rev	gctgcagtaccgcagccagaacaggtccagctc	Vif BJB
Vif BJB1 Fwd	gacctgtacagccagaccgcctgtgtgcggaag	Vif BJB1
Vif BJB1 Rev	ctggctgtacaggtcgttcatcacgaactcccatctgggg	Vif BJB1
Vif BJB2 Fwd	tcatcacctaccgctacgccatctggaagagagtgtg	Vif BJB2
Vif BJB2 Rev	gcggtaggtgatgatgatttcctctttctgcagtctggcggtcatgctgtactcggtctgccg	Vif BJB2
Vif BJB3 Fwd	gagtggaagatccagacaggcttcaccgacccc	Vif BJB3
Vif BJB3 Rev	ctggatcttccactctctggcccacacggcgtactggtaggtcagcaccagcttg	Vif BJB3
Vif BJB4 Fwd	ggacctgggctacctgatgacccctgccggcac	Vif BJB4
Vif BJB4 Rev	caggtagcccaggtccaggaagccggtctcgatggtccacactctcttcc	Vif BJB4
